# Reducing neonatal morbidity by discontinuing oxytocin during the active phase of first stage of labor: a multicenter randomized controlled trial STOPOXY

**DOI:** 10.1186/s12884-020-03331-x

**Published:** 2020-10-20

**Authors:** Aude Girault, François Goffinet, Camille Le Ray, on behalf of the collaborators of the STOPOXY trial and the Groupe de Recherche en Obstétrique et Gynécologie (GROG), Elie Azria, Tiphaine Barjat, Charline Bertholdt, Julie Blanc, Caroline Bohec, Eric Boudier, Julie Carrara, Raoul Desbriere, Muriel Doret, Charles Garabedian, Hélène Heckenroth, Gilles Kayem, Diane Korb, Gilles Levy, Georges-Emmanuel Roth, Patrick Rozenberg, Loïc Sentilhes, Eric Verspyck, Norbert Winer

**Affiliations:** 1 Université de Paris, INSERM UMR 1153, Equipe EPOPé, 123 boulevard Port Royal, 75014 Paris, France; 2 Maternité Port-Royal, AP-HP, Hôpital Cochin, FHU PREMA, F-75014 Paris, France

**Keywords:** Oxytocin, Discontinuation, Neonatal morbidity, Cesarean delivery, Labor duration, Birth experience

## Abstract

**Background:**

Oxytocin is effective in reducing labor duration, but can be associated with fetal and maternal complications such as neonatal acidosis and post-partum hemorrhage. When comparing discontinuing oxytocin in the active phase with continuing oxytocin infusion, previous studies were underpowered to show a reduction in neonatal morbidity. Thus, we aim at evaluating the impact of discontinuing oxytocin during the active phase of the first stage of labor on the neonatal morbidity rate.

**Methods:**

STOPOXY is a multicenter, randomized, open-label, controlled trial conducted in 20 maternity units in France. The first participant was recruited January 17th 2020. The trial includes women with a live term (≥37 weeks) singleton, in cephalic presentation, receiving oxytocin before 4 cm, after an induced or spontaneous labor. Women aged < 18 years, with a lack of social security coverage, a scarred uterus, a multiple pregnancy, a fetal congenital malformation, a growth retardation <3rd percentile or an abnormal fetal heart rate at randomization are excluded. Women are randomized before 6 cm when oxytocin is either continued or discontinued. Randomization is stratified by center and parity. The primary outcome, neonatal morbidity is assessed using a composite variable defined by an umbilical arterial pH at birth < 7.10 and/or a base excess > 10 mmol/L and/or umbilical arterial lactates> 7 mmol/L and/or a 5 min Apgar score < 7 and/or admission in neonatal intensive care unit. The primary outcome will be compared between the two groups using a chi-square test with a *p*-value of 0.05. Secondary outcomes include neonatal complications, duration of active phase, mode of delivery, fetal and maternal complications during labor and delivery, including cesarean delivery rate and postpartum hemorrhage, and birth experience. We aim at including 2475 women based on a reduction in neonatal morbidity from 8% in the control group to 5% in the experimental group, with a power of 80% and an alpha risk of 5%.

**Discussion:**

Discontinuing oxytocin during the active phase of labor could improve both child health, by reducing moderate to severe neonatal morbidity, and maternal health by reducing cesarean delivery and postpartum hemorrhage rates.

**Trial registration:**

Clinical trials NCT03991091, registered June 19th, 2019.

## Background

### Rationale

More than one in two women delivering in France is administered oxytocin during labor, either as part of induction of labor or because of poor uterine contractions [1]. Oxytocin is indeed effective in increasing frequency and intensity of uterine contractions and therefore in reducing labor duration [2, 3].

The most important side effect of oxytocin infusion is uterine hyper-stimulation, which has been shown to occur in more than 30% of women induced with oxytocin [4, 5]. By causing uterine hyper-stimulation, oxytocin may lead to or aggravate an abnormal fetal heart rate, contributing to neonatal acidosis. Acidosis accounts for a significant proportion of term neonatal morbidity due to related complications such as hospitalization in neonatal intensive care units, but also neonatal death or cerebral palsy in the most severe cases. In addition, the effectiveness of oxytocin in decreasing the cesarean delivery rate has not been demonstrated [6, 7]. And, its administration is potentially associated with maternal complications, such as post-partum hemorrhage [8].

Obstetric situations requiring oxytocin administration before 4 cm of dilation are induction of labor, with or without previous cervical ripening and labor dystocia, i.e. non-progression of the cervical dilation during the latent phase [9–11]. One assumption is that, once women requiring oxytocin during the latent phase enter the active phase, natural oxytocin takes over from synthetic oxytocin [12]. Thus, in the active phase, oxytocin could be discontinued, reducing exposure duration and therefore reducing the risk of complications, in particular neonatal complications such as hypoxia, without compromising the chances of vaginal delivery. It can therefore be hypothesized that discontinuation of oxytocin in the active phase of labor (from 6 cm) in women who received oxytocin in the latent phase or for an induction of labor could reduce neonatal morbidity.

### Previous published studies

Previous studies have evaluated the effect of discontinuation of oxytocin when reaching the active phase of labor on duration of the active phase of labor [13–17]. All these studies but one [15] were randomized, none used blinding, and the largest sample size was of 342 women (168 vs 174) [16]. Most of these studies, as well as the metanalysis [12] including them, showed a longer length of active phase of labor in the discontinuation of oxytocin group (in the metanalysis the mean difference was 27.65 min, 95%CI 3.94–51.36). None of these previous published trials, nor the meta-analysis combining these trials [12] showed an increase in the cesarean delivery rate in the discontinuous oxytocin group, on the contrary, the meta-analysis showed a significant decrease in the cesarean delivery rate in the discontinuous oxytocin group (9.3% compared with 14.7%; RR 0.64, 95%CI 0.48–0.87). This supports the assumption that natural oxytocin takes over from synthetic oxytocin in the active phase of labor and allows achieving vaginal delivery.

Only three studies reported the rates of postpartum hemorrhage (PPH) [14, 15, 18], one showed no differences in its rate (6.4% vs 6.3% [18]) and the other two showed a decrease in the PPH rates in the discontinuous oxytocin group without reaching statistical significance, probably due to a lack of power (16% vs 22% [14] and 1.4% vs 3% [15]). The dose and duration of oxytocin infusion is known to be associated with PPH [8], reducing these could therefore reduce the PPH risk.

Concerning neonatal morbidity, only two trials [14, 16] evaluated the impact of discontinuation of oxytocin on the rate of neonatal acidosis. One showed a two-fold reduction of neonatal acidosis in the discontinuation group (6% versus 3% umbilical arterial pH < 7.10), but the small number (*n* = 100/group) did not lead to a statistically significant difference. The other study showed no significant difference between the two groups (4% vs 5% for pH < 7.10 and 9.8% vs 7.1% for Neonatal Intensive Care Unit (NICU) admission).

## Methods

STOPOXY is a multicenter, randomized, open-label, controlled trial conducted in France.

### Setting

In 20 maternity units, in 15 cities in France.

### Population

The STOPOXY trial includes:

- Women receiving oxytocin during the latent phase of the 1st stage of labor, before 4 cm of cervical dilation, including women with an induction of labor using cervical ripening or oxytocin
- With a term pregnancy (≥37 weeks)
- Singleton pregnancy
- Fetus in cephalic presentation
- Speaking and reading French language
- Affiliated to social security
- Who have signed the consent form

Exclusion criteria are as follows:

- Women with a scarred uterus
- Fetus with a congenital abnormality
- Fetal growth retardation <3rd percentile for gestational age
- Abnormal fetal heart rate at randomization
- Maternal age < 18 years
- Women participating in another trial involving medication

### Objectives and endpoints

Our main objective is to measure the impact of a discontinuous administration of oxytocin during the active phase of the first stage of labor on the neonatal morbidity rate. Neonatal morbidity is assessed using a composite variable defined by: an umbilical arterial pH at birth < 7.10 and/or a base excess > 10 mmol/L and/or umbilical arterial lactates> 7 mmol/L and/or a 5 min Apgar score < 7 and/or admission in neonatal intensive care unit (NICU).

Our secondary objectives are to measure the impact of a discontinuous administration of oxytocin during the active phase of the first stage of labor on:

Neonatal acidosis and its severity:

- Umbilical arterial cord pH at birth less than 7.20
- Umbilical arterial cord pH at birth less than 7.10
- Umbilical arterial cord pH at birth less than 7.00
- Need for hypothermia

Other neonatal complications:

- Need for resuscitation at birth
- Transfer to a neonatal care unit
- Length of hospital stay

Mode of delivery:

- Cesarean delivery rate
- Rate of cesarean delivery for abnormal fetal heart rate
- Instrumental vaginal delivery rate
- Rate of instrumental delivery for abnormal fetal heart rate

Fetal and maternal complications during labor and delivery:

- Labor duration (active 1st stage, 2nd stage)
- Uterine hyper-stimulation, defined by periods with more than five uterine contractions in 10 min during labor
- Need for fetal scalp blood testing during labor
- Rate of fetal occipito-posterior position
- Maternal fever during labor, defined by a maternal temperature > 38 °C
- Post-partum hemorrhage, defined by an estimated blood loss > 500 mL

Birth experience:

- Post-partum women’s satisfaction using the labor agentry scale (LAS)
- A 2 months postpartum survey assessing women’s birth experience, women’s well-being using Edinburgh Postnatal Depression Scale (EPDS) and mother-child relationship, especially breastfeeding.

### Informed consent and inclusion

The initial information on the trial can be delivered before labor, during the women’s last prenatal visits by the midwife or obstetrician, during the visit at the outpatient clinic or during the birth preparation classes. The information is also distributed using posters, in the waiting rooms, emergency rooms and in the outpatient’s clinic.

Women can also be screened and informed during induction of labor or during onset of labor in case of spontaneous labor, in the labor ward. In all cases, because the cervical modifications during induction of labor are slow, especially in case of cervical ripening and during the latent phase, the delay between the final information and the inclusion appears long enough for the women to decide on their participation. All information on the chronology of the study is available in Table 1.

**Table 1:** Timeline table summarizing the chronology of the study

Time-point	Study period					
	Enrollment	Enrollment and allocation	Post-allocation			Post allocation and close-out
	D-30 (end of pregnancy)	D0 (before 6 cm)	D0 (during labor)	D0 (immediate postpartum)	D0 (after delivery in labor ward)	M2 postpartum
**Enrollment**						
Information	X					
Signature of the consent form		X				
Inclusion		X				
Randomization		X				
**Intervention**			X			
**Assessment**						
Adverse events			X	X	X	
Primary endpoints				X	X	
Secondary endpoints				X	X	X
Labor Agentry Scale Questionnaire					X	
Experience of childbirth questionnaire, EPDS						X
Breastfeeding assessment						X

Maternal pain during the onset of labor could be a concern in obtaining an informed consent. But, it has to be noted that the information on the study is mostly given before labor. Also, in the 20 participating maternity units the rate of epidural during labor is high (at least 80%). Epidural is not an inclusion criterion in the STOPOXY trial, however, most of the included women will probably receive an epidural.

The obstetrician and the midwives responsible for the labor and delivery ward are in charge of screening the includable women in the study. The inclusion is then be performed by the investigator, who will complete a computerized inclusion form, accessible 24 h on line and developed by the Cleanweb® company. The inclusion and exclusion criteria have to be met in order to include the women.

All the maternity units volunteering to participate in the STOPOXY trial are part of the Groupe de Recherche en Obstétrique et Gynécologie (GROG) network. The GROG network is a national research network affiliated with the CNGOF (Collège National des Gynécologues Obstétriciens Français).

### Oxytocin infusion protocol and randomization

During the latent phase, oxytocin is administered according to national guidelines, i.e. low dose oxytocin infusion of less than 4 mUI/min with increments every 30 min, without exceeding a 20 mUI/min flow rate [9].

Women are included and randomized in two groups before 6 cm, during the latent phase (Supplementary Material 1):

- The experimental group corresponds to discontinuation of oxytocin at the beginning of the active phase of the 1st stage of labor, i.e. oxytocin infusion is stopped beyond a cervical dilation equal or greater than 6 cm. In the experimental group, oxytocin can be re-started, if necessary, after 2 h of labor arrest (no progression of the cervical dilation and no progression of the fetal head).
- The control group corresponds to the standard care in France, i.e. when oxytocin is started during the latent phase of the 1st stage administration of oxytocin is continued during the active 1st stage and during the 2nd stage if the fetal heart rate is reassuring. As in French standard practice, the oxytocin infusion is maintained with no increments if cervical dilation progresses. If needed (abnormal fetal heart rate, uterine hyperstimulation) the infusion can be stopped.

The randomization is computerized and accessible 24 h on line. Subjects are distributed between the two groups at a ratio of 1:1. The randomization is stratified on the center and parity (nullipara/multipara). The stratification by center will allow considering the possible variations of practices between centers during labor or delivery (e.g. modalities of epidural analgesia, type of analgesic products used, etc.). Specific information on the practices is also collected in the electronic case report form (e-CRF). The stratification by parity is needed because multiparous and nulliparous women differ on several secondary endpoints such as duration of labor and mode of delivery.

Among women included in the STOPOXY trial, some will have a spontaneous labor and others an induction of labor including induction by oxytocin only or cervical ripening. Induction protocols and particularly cervical ripening methods (Prostaglandins E1, Prostaglandins E2, and mechanical methods) could differ between centers but this potential difference will be taken into account by the stratification by center.

Each change in dose of oxytocin (increments) after 6 cm of cervical dilation and until delivery will be prospectively collected and reported by the midwife or obstetrician on a specific form. The time, type of modification of infusion, reason for the change, cervical dilation and oxytocin flow will be specified.

### Choice of open-label design

The open-label design was chosen for several reasons, the main being that in case of a blinded trial, the need for un-blinding would have been too frequent. Indeed, some of the previous studies showed that during the active phase of labor, 30 to 40% of the women with a discontinued administration of oxytocin required a re-start of oxytocin after a two-hour arrest of labor [14, 15]. Another situation requiring to un-blind the randomization group is in case of non-reassuring fetal heart rate which was the case in up to 8% of the women in Saccones’ metanalysis [12]. Indeed, in the context of non-reassuring fetal heart rate (FHR) it is important for the obstetrician to know the treatment group in order to stop the oxytocin infusion to reduce uterine contractility.

The choice of this design was discussed several times on the GROG’s meetings and the final decision was to choose an open-label design. The advantages are those described above, and the bias related to the open label design will be low considering that the primary endpoint is a composite variable of objective criterions.

Apart from oxytocin administration the labor management does not differ between the two groups. The condition to re-start oxytocin in the experimental group is well described, i.e. 2 h of labor arrest (no progression of the cervical dilation and no progression of the fetal head).

### Power calculation

A prospective population-based cohort study was conducted in France in 2015 assessing French practices of induction of labor, MEDIP (MEthodes de Déclenchement et Issues Périnatales) [19]. In this study, data on oxytocin use and neonatal outcomes is available. In the French MEDIP cohort, among women receiving oxytocin under standard care (as in our control group), 8% of neonates had at least one criterion of the primary endpoint of STOPOXY trial.

We hypothesize a decrease from 8% with standard care (i.e. control group) to 5% with discontinuation of oxytocin (i.e. experimental group). Assuming this decrease, we need a sample size of 1125 women in each group, for a power of 80% and an alpha risk of 5% with the use of a bilateral test. Because some women could be included and randomized, but never reach 6 cm of cervical dilation, and thus not receive the intervention, we plan to include 10% more women. We suppose that this number will correspond to the number of women having a cesarean delivery before starting the intervention, i.e. before 6 cm. Therefore, to reach statistical significance, we would like to include 2475 women in our trial.

### Statistical analysis

#### Planned analysis

First, a descriptive analysis of the clinical characteristics of the patients at baseline will be performed. The analysis of the primary and secondary endpoints will be an intent-to-treat analysis. A per-protocol analysis, including only women who effectively received the allocated treatment will also be performed.

All tests will be bilateral with a significance level of 5%. Quantitative data will be expressed as mean ± standard deviation or median with interquartile range, and qualitative data as a percentage with a 95% confidence interval.

No interim analyses are planned in this protocol.

#### Analysis of the primary endpoint

The analysis of the primary endpoint will estimate the effect of discontinuous administration of oxytocin on neonatal morbidity defined by a composite variable. All women included and randomized in the study will be included in the analysis which will be an intent-to-treat analysis. The percentage of neonates with morbidity using the composite criteria will be compared between the two groups: discontinuous oxytocin or continuous oxytocin by a chi-square test.

#### Missing data

Children with no measure of the primary endpoint will be considered failures regardless of their randomization group. Given the composition of the primary endpoint and the usual practices in the French maternity hospitals, we think that the rate of missing data will be extremely low.

Additional analyzes will be performed on available data and after multiple imputations of missing data.

#### Analysis of the secondary endpoints

The analysis of the secondary endpoints will be done using the statistical tests adapted to the studied variables. The comparison of two means will be done by Student’s t test or if necessary by Wilcoxon tests. The comparison of percentage will be done using chi-square tests or Fisher’s exact test if necessary. All tests will be bilateral with a significance level of 5%.

#### Subgroup analysis

We plan two subgroup analyses. The first analysis will be done according to the mode of onset of labor: cervical ripening, oxytocin, spontaneous. And the second analysis will be done according to parity.

The center and parity are two criteria used to stratify our randomization. They will therefore be distributed equally among the 20 centers. These two criteria are known to be associated with the use of oxytocin and labor duration which can both play a role on our primary endpoint.

### Methods for monitoring compliance with the treatment

To avoid the contamination between the two groups because of the open-label design, the respect of the allocated group of treatment as well as the total dose and the duration of oxytocin administration will be prospectively and exhaustively monitored by clinical research assistants during the study. In case of deviations from the protocol there will be reminders to the centers and regular checks. The data collection will be performed by clinical study technicians.

## Discussion

### Potential implication of the findings

Improving management of labor represents an opportunity of improving the health of both women and infants. In most women, stopping oxytocin in the active phase may improve fetal tolerance to labor by reducing uterine hypercontractility therefore decreasing fetal heart rate abnormalities. In most severe situations, the discontinuation of oxytocin could reduce severe neonatal morbidity, which may cause neurologic damages, and moderate neonatal morbidity, which may be associated with the need of resuscitation and hospitalization. Unlike previous published studies, the STOPOXY trial, which plans to include a large sample of women, will have sufficient power to demonstrate a difference in neonatal morbidity if it exists.

In addition, we expect that the reduction of neonatal morbidity associated with oxytocin discontinuation will be associated with a reduction in maternal morbidity, in particular due to a reduction of postpartum hemorrhage which remains the leading cause of postpartum maternal morbidity.

The SPIRIT checklist can be found as Supplementary Material 2.

## Supplementary information

**Additional file 1: Supplementary Material 1**. Flow chart

**Additional file 2.** SPIRIT checklist

## Data Availability

Not applicable.

## Abbreviations

95% CI: 95% Confidence Interval
CNGOF: Collège National des Gynécologues Obstétriciens Français
GROG: Groupe de Recherche en Obstétrique et Gynécologie
EPDS: Edinburgh Postnatal Depression Scale
e-CRF: Electronic case report form
FHR: Fetal heart rate
LAS: Labor agentry scale
MEDIP: MEthodes de Déclenchement et Issues Périnatales
NICU: Neonatal intensive care unit
PPH: Postpartum hemorrhage
