# Supplementary figures and images for: Reducing neonatal morbidity by discontinuing oxytocin during the active phase of first stage of labor: a multicenter randomized controlled trial STOPOXY
Source: BMC Pregnancy Childbirth. 2020 Oct 20;20:640. doi: 10.1186/s12884-020-03331-x (PMC7576841; doi:10.1186/s12884-020-03331-x)

Supplementary material 1. Flow Chart


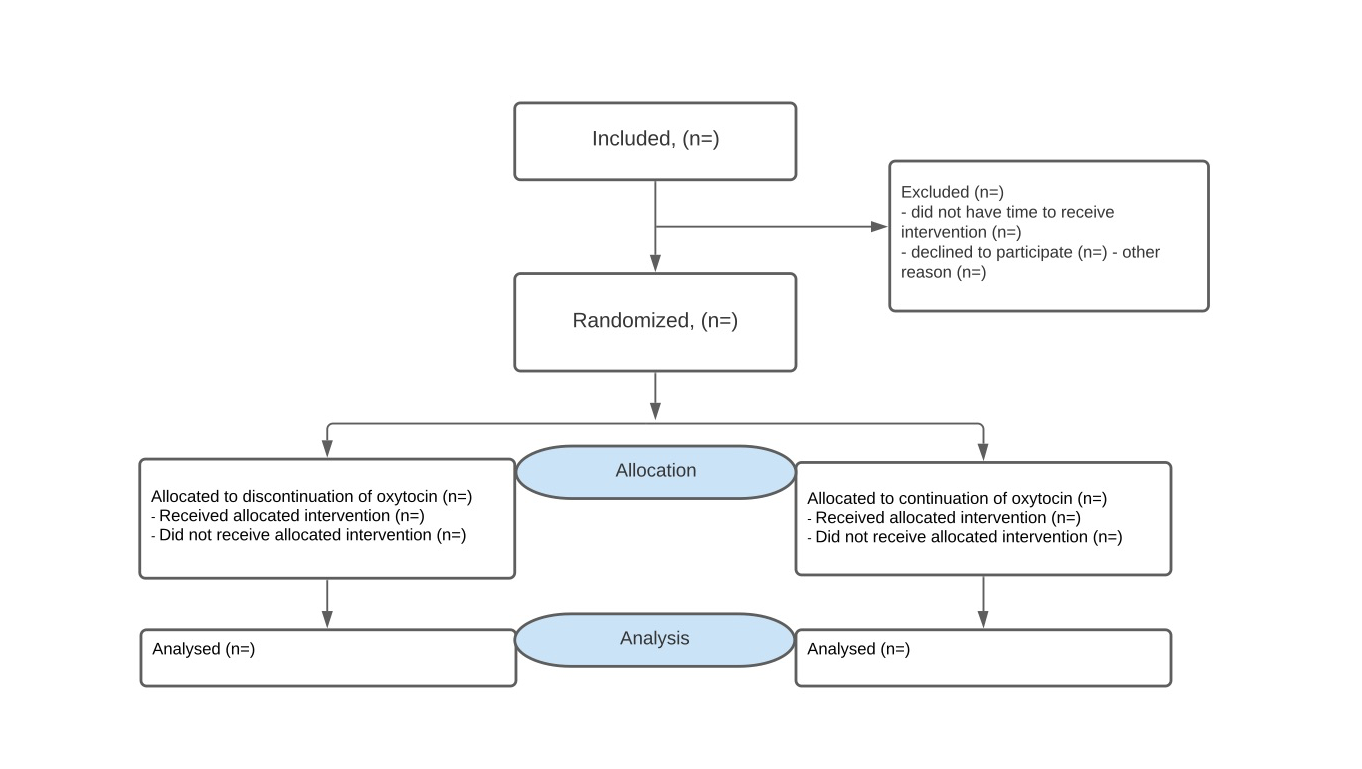

Supplement: Supplementary file 1 — Additional file 1: Supplementary Material 1. Flow chart [file 12884_2020_3331_MOESM1_ESM.docx]
